# A Homozygote Mutation in *S-Antigen Visual Arrestin SAG* Gene in an Iranian Patient with Oguchi Type One: A Case Report

**Published:** 2020-05

**Authors:** Hajar ARYAN, Atekeh BAHADORI, Dariush D. FARHUD, Marjan ZARIF YEGANEH, Haniyeh POURKALHOR

**Affiliations:** 1. Farhud Genetic Clinic, Tehran, Iran; 2. Applied Biotechnology Research Center, Tehran Medical Sciences, Islamic Azad University, Tehran, Iran; 3. School of Public Health, Tehran University of Medical Sciences, Tehran, Iran; 4. Department of Basic Sciences/Ethics, Iranian Academy of Medical Sciences, Tehran, Iran; 5. Cellular and Molecular Endocrine Research Center, Research Institute for Endocrine Sciences, Shahid Beheshti University of Medical Sciences, Tehran, Iran

**Keywords:** Congenital night blindness, Oguchi disease, *GRK1* protein, *SAG* protein

## Abstract

Oguchi disease is a rare autosomal recessive form of congenital stationary night blindness (CSNB) characterized by specific features such as golden-brown discoloration of the fundus called Mizuo-Nakamura phenomenon which is distinguishable by fundoscopy, and retinography. Clinical diagnosis is confirmed through genetic test. Two known genes in pathogenesis of Oguchi disease are *SAG* and GRK1. A 35-year-old Iranian male exhibiting the clinical features of congenital stationary night blindness, was referred to the genetic clinic of Dr. Farhud, Tehran, Iran in 2012 and examined. Ophthalmic examination including slit-lamp biomicroscopy, perimetry and funduscopy was performed. Additionally, the full-field electroretinography and molecular testing for congenital stationary night blindness were performed. Molecular genetic tests, including the analysis of *GSK1* and *SAG* genes exon-intron boundaries were performed for this patient and his family. According to the sequencing results, we did not find any mutation in *GSK1* gene. However, a new homozygote mutation at location chr2:233320735, c.517delC, p.P96LfsX28 was identified in exon four of *SAG* gene. This deletion causes a frame shift mutation, and premature stop codon that results in deletion of about 281 amino acid residues of S-antigen visual arrestin protein (from entire C-terminal). This mutation was also found in patient’s parents and one of his sister as heterozygote form. This is the first molecular evidence for *SAG* gene mutation in an Iranian family affected with Oguchi disease type 1. The identification of the new c.517delC, p.P96LfsX28 mutation in this family with Oguchi disease can confirm the pathogenicity of this variant.

## Introduction

Congenital Stationary Night blindness (CSNB) is a group of diseases identified by a non-progressive disorder in retina (1). CSNB is caused by a mutation in the component (photo transmission) or a mutation in the signaling pathway from the outer retina to the internal one (1).

Oguchi disease is a rare autosomal recessive type of congenital stationary night blindness. In this disease usually, other visual functions such as visual acuity, visual field, and color vision, are normal. A typical feature of Oguchi disease is a golden or gray-white discoloration of the fundus that disappears in the dark-adapted state and reappears shortly after the onset of light (Mizuo phenomenon, or Mizuo-Nakamura phenomenon). The course of dark adaptation of rod photoreceptors is extremely retarded, whereas that of cones appears to proceed normally. Oguchi disease-1 (CSNBO1) is caused by homozygous or compound heterozygous mutation in the S-antigen visual arrestin gene (*SAG*) on chromosome 2q37.1(2, 3). S-arrestin, or S-antigen, is a major soluble photoreceptor protein that is involved in desensitization of the photoactivated transduction cascade. It is expressed in the retina and the pineal gland and inhibits coupling of rhodopsin to transducin in vitro. The S-arrestin or S-antigen protein, consists of 405 amino acids and 48 kDa protein, is encoded by a gene that contains 21 exons (4–7). Different mutations in this gene have been identified in Oguchi’s disease, and Retinitis Pigmentosa (recessive form) (2, 8).

Oguchi disease-2 (CSNBO2) is caused by mutation in the rhodopsin kinase gene (*GRK1*) on chromosome 13q34. This gene encodes a member of the guanine nucleotide-binding protein (G protein)-coupled receptor kinase subfamily of the Ser/Thr protein kinase family. The protein phosphorylates rhodopsin and initiates its deactivation. Genetic mutations in *GRK1* are known to cause Oguchi disease 2 (also known as stationary night blindness Oguchi type (2–9).

Proteins encoded by *SAG* and *GRK1* genes are components of the photo transmission pathway in photo receptor cells (4, 9). It has been shown that Oguchi disease is more prevalent in the Japanese population than in other populations (4, 9–11).

This report was conducted to molecular genetic investigation in an Iranian family affected with Oguchi disease type 1.

## Case Presentation

The patient, a 35-year-old Iranian male was referred to Farhud’s Genetics Clinic, Tehran, Iran in 2012. The chief complaint of this patient was related to the blindness at that time. After clinical examinations, he was diagnosed with Oguchi disease. In the medical history of the patient, his parents were first cousin and he had two apparently healthy sisters. Written informed consent was taken from the patient.

After obtaining whole blood samples from the patient and his family members, DNA was extracted using standard salting out/proteinase K method, and examined for genes that involved in Oguchi disease, including *GRK* and *SAG* genes using PCR and sequencing methods.

**Fig. 1:**
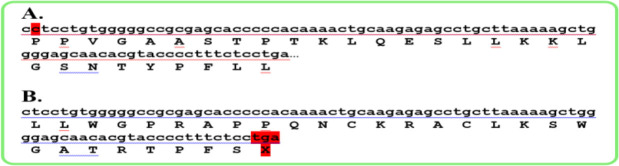
A) A part of exon four sequence of *SAG* gene, without C nucleotide deletion (location chr2:233320735, c.517delC, p.P96LfsX28) B) The same part of exon four of *SAG* gene, with C nucleotide deletion which is resulted in a frame shift mutation and premature stop codon in this gene

**Fig. 2:**
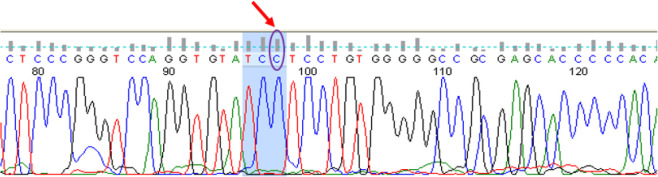
The normal sequencing result of *SAG* gene exon four in a completely healthy sister, without a heterozygote C nucleotide deletion (location chr2:233320735, c.517, p.P96LfsX28). The C nucleotide showed by arrow in this figure, was deleted in patient and parents

**Fig. 3:**
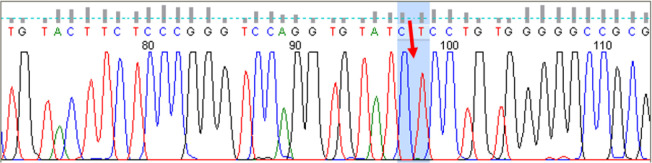
The sequencing result of *SAG* gene exon four in affected patient, with a homozygote C nucleotide deletion (location chr2:233320735, c.517delC, p.P96LfsX28)

The sequencing results of *GRK* gene exon-intron boundaries did not show any mutations in this patient, but the sequencing of the *SAG* gene indicated a new homozygous mutation in exon four. This mutation was a C nucleotide deletion at codon 96, location chr2:233320735, c.517delC, p.P96LfsX28. This deletion causes a frame shift mutation, and premature stop codon in exon four of *SAG* gene (Figs. 1–3).

**Fig. 4:**
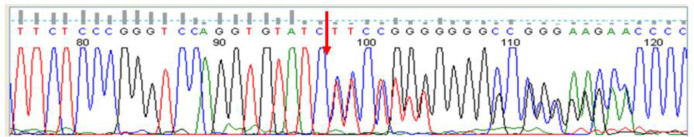
The sequencing result of *SAG* gene exon four in parents and one of patient’s sister, with a heterozygote C nucleotide deletion (location chr2:233320735, c.517delC, p.P96LfsX28)

The nucleotide sequence of the 4th exon of the *SAG* gene in his parents showed that they were both heterozygotes for C deletion in codon number 96 (Fig. 3) and as mentioned above, the patient was homozygote for these mutations. One of her sister was also heterozygote for this new mutation. The results of the other sister’s patient showed that she is healthy for deletion of codon No. 96 of the *SAG* gene mutation (Fig. 4).

## Discussion

There are three forms of congenital Stationary Night Blindness (SNB), including Constant congenital blindness (CSNB), Fondus albipunctatus, and Oguchi disease (12). Oguchi disease is a rare autosomal recessive congenital disease. Its SNB shape is detected by a special grayscale or grayscale color that returns to normal after a long time darkness (13).

Oguchi disease is a rare condition, which approximately 50 cases have been reported to date. The disease was first discovered in Japan, where the highest incidence was reported, but some cases have also been diagnosed in Europe, the United States, Pakistan, and India. As mentioned above, mutations in *SAG* and GSK can cause ogushi disease type 1 and 2, respectively. It is noteworthy that some of the mutations in the *SAG* gene are associated with UGC and retinitis pigmentosa (RP) in a family. Some *SAG* mutations cause RP disorder (2, 3, 7, 14). The S-antigen, or arrestin, is a photoreceptor-specific soluble protein that plays an important role in quenching the phototransduction cascade by inactivating phosphorylation-activated rhodopsin

Clinical diagnosis of blindness and determination of the Mizuo-Nakamuro phenotype are examined by fundoscopy and electroretinography (ERG). Finally it is confirmed by genetic testing (16). Electromechanical changes are important in the definitive diagnosis of Oguchi disease. The contrast and electro controls are important in understanding the complex mechanism of the disease (15).

Electroretinograph (ERGs) and dark curves are unusual in patients with congenital hereditary blindness. The appearance of the eye can be varied, but includes visual dimming and refractive error (usually myopia, sometimes double vision), nystagmus, strabismus, and the appearance of the fundus that have changed (16).

A few centuries ago, people who were unable to see at night. Got embarrassed when the weather was getting dark, and if the mentioned people experienced that situation out of house or outside and in the dark when they were dawning, and when they fell in the middle of the night, they were straightening their heads or hugging their hands (17).

This situation is very different today. With the artificial lighting that is everywhere, we are able to see the colors. During the night, a group of specialized, cylindrical vision cells are not essential for many night activities. Night blind people may not complain of this condition. They probably do not recognize this problem (14).

A study by Mehmet Yasin Teke and his colleagues in Turkey in 2016 was conducted on a Turkish family with the disease of Oguchi. In their study patients diagnosed with the Oguchi clinical features were identified, the genetic study revealed that a new mutation of *GRK1* (C.923T> C) caused Oguchi disease in all siblings. This study was able to detect a missense mutation, a new meaning in the *GRK1* that affected members of a Turkish family (18).

Nadia K. (Waheed and colleagues) was the first reported case of type 1 Oguchi disease in Pakistan, which resulted in the identification of a nonsense mutation (c 916 G> T: PGLU306) in *SAG* gene. The hematologyical and neurological anomalies are not accompanied by different variants of *SAG*. The mutation analysis was performed by determining the sequence of 2 candidate antigen genes (*SAG*s) associated with Oguchi type 1 and rhodopsin kinase 1 associated with Oguchi type 2 .In addition, the C677T variant was investigated in the methylene tetrahydrofolate reductase gene in the family to show the possibility of hypercysteinemia in patients. Determination the governor of *SAG* and *GRK1* showed that a homozygosity of a meaningful mutation (c.916G> T; PGlu 306) did not affect its siblings and brothers (8).

In another study, 5 of 6 unrelated Japanese patients with Oguchi disease was identified a homozygous deletion of nucleotide 1147 in codon 309, resulting in a premature stop codon and functional null alleles (5). In 2 unrelated Japanese patients with Oguchi disease, a compound heterozygosity and homozygosity was found, respectively in the *SAG* gene (11).

In two Indian brothers with Oguchi disease identified homozygosity for a nonsense mutation in the *SAG* gene. One of them showed pigmentary retinal degeneration that similar molecular pathologic events might be responsible for pigment formation in both Oguchi disease (19).

In a 15-year-old Pakistani girl with typical Oguchi disease, identified homozygosity for a nonsense mutation in the *SAG* gene that was heterozyge in her unaffected mother and four other unaffected relatives and was not found in a healthy control panel from the same population. The authors stated that this was the first Pakistani patient with Oguchi disease due to a *SAG* mutation (20).

Regarding the fact that any special treatments has not yet found for this disease, the necessity of its recognition is of great importance for patients, since if the disease is mistakenly diagnosed as problems like low vision, there will cause serious difficulties while driving, or in areas where Light dimmers, such as low light restaurants, cinema and theater.

## Conclusion

This is the first report of a new homozygote c.517delC, p.P96LfsX28 frameshift mutation in the N-terminal domain of Arrestin (or S-antigen) protein in an Iranian family affected with Oguchi disease type1.

## Ethical considerations

Ethical issues (Including plagiarism, informed consent, misconduct, data fabrication and/or falsification, double publication and/or submission, redundancy, etc.) have been completely observed by the authors.
